# Protein Domain of Unknown Function 3233 is a Translocation Domain of Autotransporter Secretory Mechanism in Gamma proteobacteria

**DOI:** 10.1371/journal.pone.0025570

**Published:** 2011-11-01

**Authors:** Ananth Prakash, S. Yogeeshwari, Sanchari Sircar, Shipra Agrawal

**Affiliations:** 1 Institute of Bioinformatics and Applied Biotechnology, Bangalore, India; 2 BioCOS Life Sciences Private Limited, Bangalore, India; Russian Academy of Sciences, Institute for Biological Instrumentation, Russian Federation

## Abstract

*Vibrio cholerae*, the enteropathogenic gram negative bacteria is one of the main causative agents of waterborne diseases like cholera. About 1/3^rd^ of the organism's genome is uncharacterised with many protein coding genes lacking structure and functional information. These proteins form significant fraction of the genome and are crucial in understanding the organism's complete functional makeup. In this study we report the general structure and function of a family of hypothetical proteins, Domain of Unknown Function 3233 (DUF3233), which are conserved across gram negative gammaproteobacteria (especially in Vibrio sp. and similar bacteria). Profile and HMM based sequence search methods were used to screen homologues of DUF3233. The I-TASSER fold recognition method was used to build a three dimensional structural model of the domain. The structure resembles the transmembrane beta-barrel with an axial N-terminal helix and twelve antiparallel beta-strands. Using a combination of amphipathy and discrimination analysis we analysed the potential transmembrane beta-barrel forming properties of DUF3233. Sequence, structure and phylogenetic analysis of DUF3233 indicates that this gram negative bacterial hypothetical protein resembles the beta-barrel translocation unit of autotransporter Va secretory mechanism with a gene organisation that differs from the conventional Va system.

## Introduction

Domain of Unknown Function (DUF) 3233 (PFAM: PF11557) is a family of uncharacterised hypothetical proteins conserved among gram negative gammaproteobacteria. Representative members of this domain include marine bacteria from genus Vibrio, Shewanella, Colwellia and Alcanivorax of which *Vibrio cholerae*, *Vibrio parahaemolyticus*, *Vibrio splendidus* and *Vibrio vulnificus* are pathogenic to human and aquatic life. *Vibrio cholerae* causes seasonal outbreaks of cholera of epidemic proportions in developing countries with high mortality rates [1]. The enterotoxins produced by the bacteria after colonising the host small intestine disrupts the ion transport by the intestinal epithelial cells causing outflow of large volumes of fluids into the intestine leading to watery diarrhoea, dehydration and in severe cases, death [1] [2].

Significant fraction of genomes of Vibrio species lack structure function annotation and most of these gene products are classified as hypothetical proteins or domains of unknown function [3]. The PFAM [4] database in its 24^th^ release lists about 3000 DUF families. Many of these DUF families are kingdom specific (DUF2883, DUF3328, DUF3329), limited/shared between kingdoms (DUF1497, DUF3609) or restricted/specific to certain organisms (DUF1196, DUF2667). The specific and ubiquitous nature of these domains suggests their functional importance in organism specific niches or a common biological role.

Identifying homologous protein families through sequence based search marks the first step in the annotation of DUFs, providing an initial broad picture of the protein's probable family and function. Sequence homology search becomes increasingly powerful when we advance from normal sequence-sequence based searches to methods that uses profile or HMM information like HHsenser [5], which increases the efficiency of finding remote homologues. *In silico* structure prediction methods together with sequence similarity detection methods assist the annotation of fold-function space. Fold recognition methods like I-TASSER [6] help predict the 3 dimensional (3D) structure and functions of proteins that share low sequence identity with other known structures.

In this study we analyse the sequence and structural characteristics of DUF3233 using computational approaches and try to infer various properties of this domain. Sequence search by HHsenser identifies similarity with the beta-barrel translocation unit of autotransporter Va secretory proteins. Sequence homology combined with secondary structure prediction indicates a beta-barrel domain of 12 beta-strands. The predicted 3D model from I-TASSER shows the structure with an overall beta-barrel topology with an N terminal helix running along the central barrel axis perpendicular to the 12 antiparallel strands that form the barrel. Amphipathicity and membrane barrel discrimination analysis suggest the domain is a potential outer membrane gram negative beta-barrel protein.

Autotransporter translocation units belong to the transmembrane beta-barrel fold in SCOP database [7], defined by a beta-barrel of 12 to 14 antiparallel strands with an N terminal helix perpendicular to the barrel. Finally with the analysis of genomic context of DUF3233 we could infer that this outer membrane beta-barrel translocation domain has a gene organisation that is not typical of the autotransporter Va secretory mechanism.

## Results

### Sequence based characterization of DUF3233 as autotransporter β-domain protein

Sequence search for homologues with PSI-BLAST using a representative query, *Vibrio cholerae* DUF3233 (RefSeq: NP_232949) against the NCBI nr database with a threshold 0.005, reached convergence at the 4^th^ iteration. The resulting sequences identified were hypothetical proteins conserved among gram negative proteobacteria. For improved search and better coverage of homologous sequence space, information from aligned regions of DUF3233 sequences in the form of a multiple sequence alignment profile was queried with HHsenser. From the resulting sequences in the permissive alignment list we were able to infer homology between DUF3233 and the outer membrane beta-barrel translocation domain of autotransporter proteins. DUF3233 shares sequence similarity with outer membrane beta-barrel domain of *Ochrobactrum intermedium* autotransporter (e-value 1E-34, 95% coverage, 22% identity), *Rhizobium leguminosarum* adhesin autotransporter (e-value 2E-29, 94% coverage, 18% identity) and *Yersinia aldovae* AidA adhesin autotransporter (e-value 2E-26, 89% coverage, 18% identity). Interestingly, a number of gram negative hypothetical proteins were picked up as potential homologues, which showed fair amount of similarity to the autotransporter beta-domain (Table S1).

Position specific scoring matrix (PSSM) profile based discrimination analysis using TMBETADISC-RBF [8], predicts the outer membrane beta-barrel nature of DUF3233. To confirm homology with autotransporter protein family we queried DUF3233 sequences against putative outer membrane proteins (OMPs). A pairwise hidden Markov model (HMM) search by HHomp [9] identifies DUF3233 as an OMP. *V. cholerae* DUF3233 shares homology with HHomp cluster 12.1.6 (96% probability). This cluster comprises profile HMMs of autotransporter sequences whose 12 stranded beta-barrel transmembrane domains conform to the translocation unit of autotransporter NalP [9]. DUF3233 sequences from Colwellia (94% probability), Shewanella (96% probability) and Ferrimonas (95% probability) were all found to share homology with autotransporter protein family.

### DUF3233 is a solitary outer membrane autotransporter β-barrel domain

Proteins targeted for transport across membranes posses leader sequence or signal peptide at their N-terminus, which directs translocation. We analysed DUF3233 sequences using a combination of artificial neural networks and HMMs implemented in SignalP [10] to predict the presence and location of signal peptide cleavage sites. SignalP identified the presence of N-terminal signal peptide of an average length of 23 amino acid residues having a positively charged amino terminal followed by a hydrophobic region and hydrophilic carboxy terminal. Signal peptides are cleaved from the exported protein by specific proteases called signal peptidases (SPases) [11]. Prediction of cleavage mechanism of these signal sequences by LipoP [12] identifies SPase 1 target site, indicating DUF3233 might be a non-lipoprotein.

We browsed DUF3233 genomic region of all representative organisms with STRING [13] to look for possible gene fusion events with other domains and found no such occurrence. DUF3233 is a single domain protein found on the small chromosome 2 in Vibrio species with an upstream gamma-glutamyltranspeptidase (GGT) or response regulatory protein transcribed in one potential operon (Table 1). These upstream proteins lack the N-terminal signal sequence for inner membrane transport and analysis through SecretomeP [14] indicates that these proteins are not exported through non-classical secretory system. Gene organisation of DUF3233 therefore suggests a solitary translocation unit with an absent upstream secretory protein.

**Table 1:** Genomic context of DUF3233: Proteins upstream of DUF3233 in various representative organisms are organised in operons and have a probable pathogenic role.

DUF3233 Gene	Organism	Upstream protein	DOOR Operon ID*
*Fbal_1102*	*F. balearica* DSM 9799	OmpA domain protein transmembrane region-containing protein	-
*VCA0559*	*V. cholerae* O1 biovar El Tor str. N16961	Gamma-glutamyltranspeptidase, putative	-
*VC0395_0494*	*V. cholerae* O395	Putative gamma-glutamyltranspeptidase	329019
*VPA0926*	*V. parahaemolyticus* RIMD 2210633	Putative regulatory components of sensory transduction system	88118
*VEA_000122*	*Vibrio sp.* Ex25	Response regulator	-
*VV2_0321*	*V. vulnificus* CMCP6	Response regulator	80840
*VVA0816*	*V. vulnificus* YJ016	Putative regulatory components of sensory transduction system	102681

*For few genomic strains which are not represented in DOOR v2.0, operon ids are left blank.

### Structure based validation of DUF3233 as transmembrane β-barrel domain of autotransporter proteins

**Figure 1:**
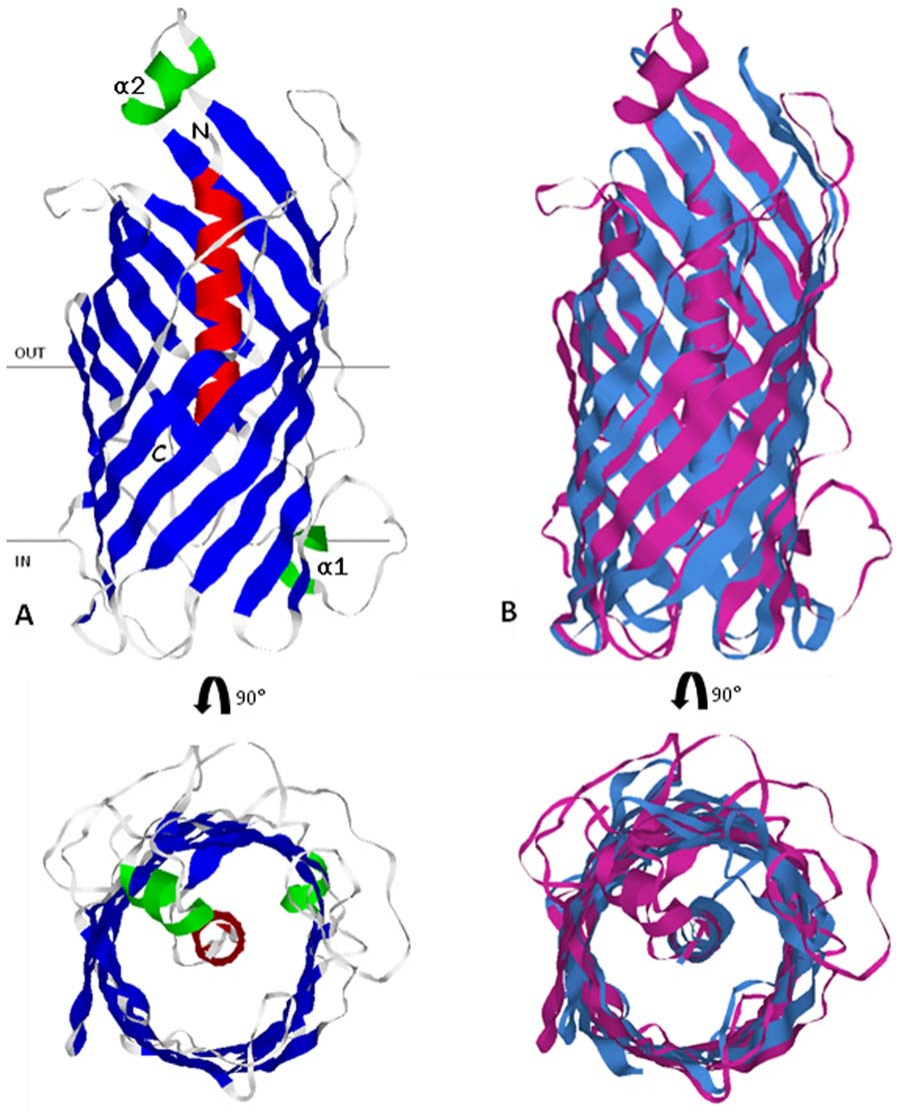
Predicted 3D model of DUF3233. **A**) The predicted structure resembles the beta-barrel translocation unit of autotransporter proteins. The 12 beta-strands (blue) orient in anti-parallel fashion enclosing the N-terminal helix (red), the two additional helices α1 and α2 are shown in green. **B**) Superimposed structures of DUF3233 (magenta) and NalP translocation unit of *N. meningitidis* 1uyn_x (blue). Figures were generated with Rasmol.

**Figure 2:**
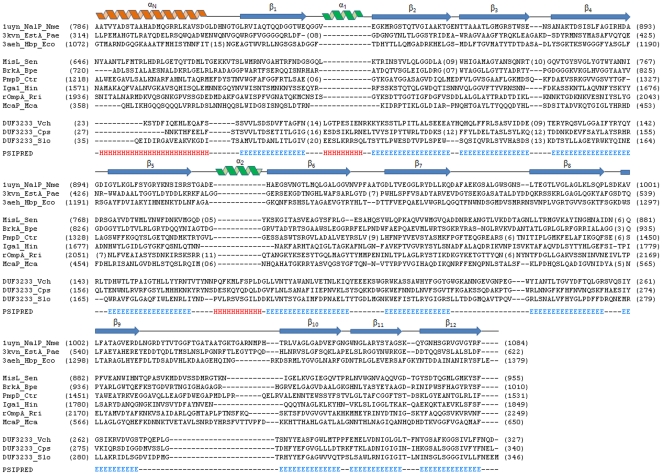
Sequence alignment of DUF3233 and translocation units of select autotransporters. Secondary structure diagram for NalP is shown at the top. The two extra helices (α_1_ and α_2_) present among DUF3233 proteins are shown in green. DUF3233 and autotransporter sequences representing each cluster are used in the alignment. Nme_*Neisseria meningitidis*, Pae_*Pseudomonas aeruginosa*, Eco_*Escherichia coli*, Sen_*Salmonella enterica*, Bpe_*Bordetella pertussis*, Ctr_*Chlamydia trachomatis*, Hin_*Haemophilus influenzae*, Rri_*Rickettsia rickettsii*, Mca_*Moraxella catarrhalis*, Vch_*Vibrio cholerae*, Cps_*Colwellia psychrerythraea*, Slo_*Shewanella loihica*.

**Figure 3:**
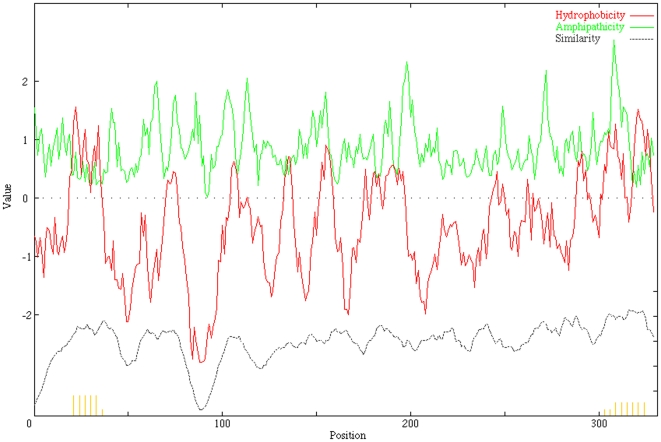
Average hydropathy, amphipathicity and similarity plot of 18 DUF3233 representative sequences. The plot shows 12 hydrophobic (green) and amphipathic (red) peaks corresponding to possible transmembrane beta-strand forming segments The plot was generated using AveHAS program.

DUF3233 sequence based PSI-BLAST search for proteins with known structures (72,386 structures in PDB as of April 2011) fetched results with a maximum alignment length covering 51 residues. Secondary structure assignment by PSIPRED [15] predicts an N-terminal α-helix (α_N_) followed by 12 consecutive β-strands (β_1_–β_12_) interspersed by two short turns of α-helices α_1_ and α_2_ predicted to occur between β_1_–β_2_ and between β_5_–β_6_ respectively. With no suitable template with significant sequence homology available, we used the fold recognition algorithm implemented in I-TASSER to predict a 3D model of DUF3233. The translocation unit of NalP from *N. meningitidis* (PDB: 1UYN_X, 15% identity, 85% coverage, normalised Z-score above 1) was identified by I-TASSER in the top four threading templates to model *V. cholerae* DUF3233. The predicted structure of *V. cholerae* DUF3233 (Figure 1) resembles the beta-barrel translocation unit of autotransporter proteins, aligning over 75% structurally equivalent positions with the template and an RMSD of 2.3. The domain has an N-terminal α-helix running along the central axis surrounded by beta-barrel formed by twelve anti-parallel beta-strands. Predicted strand assembly within the outer membrane shows the carboxy and amino terminal of the beta-barrel point towards the periplasmic space, the central helix is oriented such that its N-terminal is pointed towards the external environment. Secondary structure based sequence alignment of DUF3233 with the translocation unit of autotransporters shows a similar domain organisation (Figure 2). Using alignment of DUF3233 sequences the average hydropathy, amphipathicity and similarity plots was generated with AveHAS [16]. Figure 3, shows 12 hydrophobicity and amphipathicity peaks with an average stretch of 10 to 15 residues per peak that may form transmembrane beta-strands.

### DUF3233 is evolutionarily linked to the autotransporters

**Figure 4:**
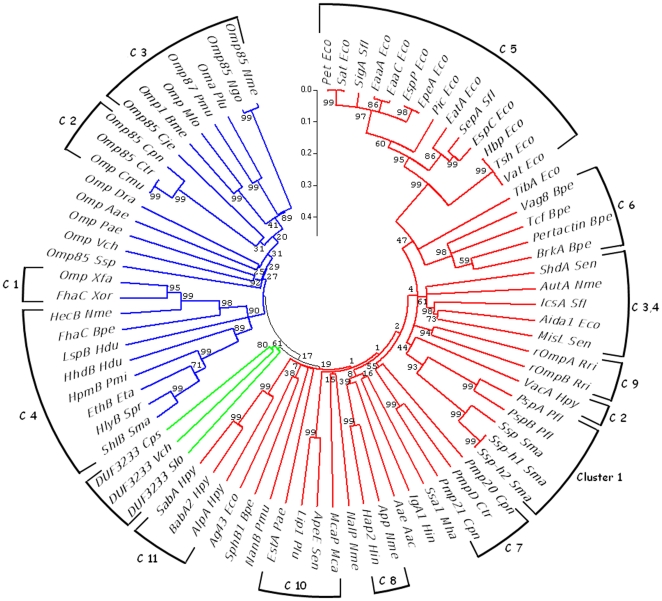
Bootstrap phylogenetic tree of DUF3233 with Va and Vb family proteins. 51 Va family (red), 25 Vb family (blue) translocation unit and 3 DUF3233 (green) representative sequences were used to generate a 1000 replicate bootstrap phylogenetic tree. Eukaryotic Vb sequences representing clusters 1 and 2 were not considered in the analysis. Sequences of each family are grouped into previously reported clusters (C 1 to 11 and C 1 to 4); DUF3233 sequences form a new cluster of the autotransporter family. Numbers at the node indicate neighbour-joining bootstrap percentages. Phylogenetic analyses were conducted in MEGA4.

To infer evolutionary relation with type V secretory proteins, we analysed DUF3233 representatives with members of both Va and Vb family (Table S2). The third type of proteins found in the type V secretory system, type Vc or AT-2 proteins, which are characterised by trimeric C-terminal beta-barrel [17] were not considered for phylogenetic analysis. The inferred phylogenetic tree (Figure 4) classifies members of the two families into two separate clans. Proteins are grouped into clusters with similar function, architecture and organism type as analysed in [18] and [19]. DUF3233 family sequences though related to autotransporters form a distinct group from the main autotransporter clan indicating that these domains represent new cluster of autotransporters.

## Discussion

A vital part in the survival and adaptation mechanism of bacteria lies in the constant interaction with their extra-cellular environment. Bacteria secrete a wide range of molecules into the extra-cellular milieu that includes enzymes, which break down carbohydrates, proteins and lipids, and virulence factors such as adhesins and toxins by those involved in pathogenesis. Transport of these molecules is mediated by protein complexes through conserved secretory pathways. Of the 6 types of secretory mechanisms known in gram-negative bacteria (type I to type VI), type V represents the simplest transport system. Proteins of the type V secretory system fall under the autotransporter (Va), two partner secretion (Vb) and the AT-2 (Vc) families, and share a similar domain organisation: an N-terminal signal peptide for inner membrane translocation followed by a passenger protein which is normally a virulence determinant and a C-terminal translocation unit for transporting the upstream passenger protein [18].

Proteins of the autotransporter (Va) family were the first to be described [20] and form the largest representation of this system [19]. Autotransporters export a wide range of toxins and enzymes [21] to the cell surface or secrete them into the external environment. The passenger domain and translocation unit of autotransporters are both expressed as a single polypeptide [20] making the translocation unit highly specific and committed for transporting only the upstream passenger. Solved experimental structures of the autotransporter translocation unit [22]–[24] show that they all possess a similar structure, a beta-barrel of 12 antiparallel strands with a central N-terminal helix running along the barrel axis. Proteins of the two partner secretion (Vb) are widely distributed and follow a similar mode of function, transporting cytolysins, adhesions and metalloproteases [19]. The secreted exoprotein and the transporter unlike the Va proteins are not linked but, are expressed as two separate proteins transcribed in a single operon [25]. Vb transporters are predicted to have a multidomain architecture [26] and a relatively wider barrel made of 16 [27] to 20 [28] beta-strands. The newly discovered AT-2 family (Vc) [29] represents proteins secreted via a homotrimeric mechanism [30]. Proteins secreted through this system are mainly implicated in virulence [31]. With a domain organisation similar to that of autotransporters, the system functions with the coming together of three individual proteins each complete with an N-terminal signal peptide, a passenger unit and four beta-strand domain at the C-terminal which makes a complete closed 12 stranded beta-barrel translocation unit upon trimerisation [31] [32].

The present work describes sequence and structure based characterization of proteobacteria DUF3233 as a beta-barrel transmembrane domain of autotransporter proteins. DUF3233 packs an average 312 amino acid residues (including N-terminal signal peptide) and is currently classified as a domain of unknown function.

DUF3233 is encoded as a single domain protein, homologous to the translocation unit of autotransporters. One aspect of DUF3233 that distinguishes it from other main class autotransporters is that it lacks a covalently linked N-terminal passenger domain, to which C-terminal translocation units of all autotransporters are committed to transport. Few autotransporter representatives of two-polypeptide architecture [19] might suggest the secretion of co-transcribed upstream proteins similar to the TPS system, but considering the cytosolic nature of upstream proteins, extracellular translocation seems unlikely.

Few representative members from the Vibrio genus express DUF3233 and upstream putative GGT or response regulatory proteins in a single operon (Table 1). Over expression of GGT [33] and GGDEF domain proteins [34] [35] are implicated in pathogenesis. The prokaryotic GGT is shown to be a major factor in the colonisation of gut and gastric mucosa [36] [37]. The upstream response regulators are two-domain proteins with an N-terminal CheY-like regulatory and a conserved C-terminal GGDEF effector domain, which is responsible for eliciting pathogenic response through cyclic di-GMP mediated exopolysaccharide synthesis and biofilm formation [38]. Genes encoding virulence products in *V. cholerae* are organised in clusters or operons [39], and since gene encoding DUF3233 is located among virulent genes, the possibility of the involvement of DUF3233 in pathogenesis cannot be overlooked.

The translocation units of autotransporters exhibit conserved amino acid consensus motif at their carboxy terminus, the barrel closing beta-strand displays alternate arrangement of polar and hydrophobic residues terminating with a conserved aromatic amino acid at the barrel terminus which is usually a phenylalanine or a tryptophan [40]. Hendrixson *et al.*, [41] demonstrated the importance of C-terminal consensus motif on the viability of *H. influenzae* Hap translocation unit. Deletion of terminal 12 residues proved detrimental to the outer membrane localisation; while the stability and/or outer membrane localisation of the translocation unit was affected with the deletion of all three terminal residues, point mutations of these residues showed no effect on the outer membrane localisation or secretion of the mature protein [41]. DUF3233 displays consensus pattern at its C-terminal that resembles the conserved motif found among autotransporters discussed above (Figure S1). A stretch of alternating polar and hydrophobic residues precedes the terminal beta-strand having a hydrophobic segment and a conserved ‘terminal’ phenylalanine or a tyrosine residue. Interestingly the extreme carboxy terminus harbours three conserved polar residues [N/D][Q/E][D/E] after the ‘terminal’ F/Y. Secondary structure and predicted models of DUF3233 show the hydrophilic residues of the “polar tail” are not part of the terminal beta-sheet, but instead form a short overhang pointed towards the periplasm. As yet, we do not know the significance and possible role of these tail polar residues on the outer membrane localisation and stability.

DUF3233 exhibits certain features that are in common with the translocation units of type Va secretory proteins and yet possesses characteristics that are not typical to the proteins of the above system. DUF3233 represents a translocation unit that is devoid of a secretable passenger unit. Considering its location in the representative genomes alongside other virulence genes, we hypothesize that this domain is involved in pathogenesis. However, the mechanism apparently looks new and different than a typical type Va secretion system.

Our study on the proteobacterial protein DUF3233 with a combination of methods like sequence similarity searches, outer membrane beta-barrel discrimination, phylogenetic analysis, and fold recognition has led us to a consensus at annotating fold and function to this domain.

Sequence similarity search suggests that DUF3233 has remote homology with the translocation unit of autotransporter proteins of the type V secretory system. The domain's outer membrane beta-barrel nature was further emphasised by signal peptide, outer membrane beta-barrel discrimination and amphipathicity analysis. Secondary structure prediction and alignment with translocation units, and inputs from the predicted model suggests that DUF3233 and the translocation unit of autotransporter proteins share a similar domain organisation.

Drawing a consensus from various *in silico* prediction methods it appears that DUF3233 is a cluster of remote homologues of autotransporters. This is the first report of an autotransporter like protein family in Vibrio species, and though within the realm of bioinformatics we were able to infer its probable family and fold, the pathogenic mechanism still remains to be explored and seeks further experimental studies.

## Materials and Methods

### Sequence similarity search

20 DUF3233 genes from NCBI comprising one sequence each from Aliivibrio, Colwellia and Ferrimonas, five sequences from Shewanella and twelve from Vibrio species were fetched using their corresponding RefSeq ids. YP_001366070 and YP_001555810 were excluded because of their short domain size and a total of 18 DUF3233 sequences were included in this study. The signal peptides of DUF3233 sequences have not been included in various analyses of this study unless mentioned. PSI-BLAST [42] profile-sequence search was used to probe homologous protein families against NCBI nonredundant (nr) database with default parameters. ClustalW multiple sequence alignment profile of DUF3233 protein sequences was taken in as input for HHsenser to search the nr database with a threshold e-value of 0.001 and default parameters for improved homolog coverage. TMBETADISC-RBF [8], PSSM profile based discrimination of beta-barrel OMPs from other folding types like globular and membrane proteins was used to assess the outer membrane nature of DUF3233. DUF3233 sequences were queried against HHomp database [9] to detect homology with other known OMPs.

### Signal peptide and Genomic context analysis

The presence of N-terminal signal peptide and the putative cleavage sites were predicted with SignalP 3.0 [10]. Using LipoP 1.0 [12] the signal peptide sequences were checked for lipoprotein signal peptide signatures that differentiate them from other signal peptides and subsequently cleavage by signal peptidase II from signal peptidase I. SignalP 3.0 and SecretomeP 2.0 [14] were used to determine inner membrane transport of proteins upstream of DUF3233 via Sec dependent or other non classical secretory pathways. STRING (version 8.3) [13] and DOOR (version 2.0) [43] were used for gene neighbourhood and operon analysis.

### Structure prediction and transmembrane β-barrel analysis

Secondary structure assignments were made using PSIPRED [15]. 3D structure of *V. cholerae* DUF3233 (RefSeq: NP_232949) was predicted using I-TASSER fold recognition method [44]. The predicted structure was superimposed with the template using TopMatch [45] and visualised with Rasmol [46]. The WHAT [47] program was used to predict hydropathy and amphipathicity using sliding windows of 13, 15, 17 residues and an angle of 100° for α-helix and 180° for β-strand. Multiple sequence alignment profile was used to plot the average hydropathy, amphipathicity and similarity plot using the AveHAS [16] program. Orientation of the domain within the outer membrane was predicted using the Viterbi method implemented in PRED-TMBB [48]. Secondary structure based sequence alignment of DUF3233 family and with the representative autotransporter translocation units was done with inputs from ClustalX [49], ProbCons [50], Ali2D [51] and the alignment was adjusted manually.

### Phylogenetic analysis

Amino acid sequences of translocation unit of autotransporters, and pore forming beta-domain of two-partner secretion (TPS) family members, reported in [18] and [19] were used for phylogeny analysis. Three representative DUF3233 sequences from *V. cholerae* (RefSeq: NP_232949), *C. psychrerythraea* (RefSeq: YP_269983) and *S. loihica* (RefSeq: YP_001095898) along with beta-barrel domain sequences of the Va, Vb secretory systems were analysed with MEGA 4 [52]. Pairwise distances were calculated and the phylogenetic tree of the aligned sequences was generated using minimum evolution method. A bootstrap test of phylogeny was performed with p-distance model on the inferred evolutionary tree and a consensus bootstrap tree was generated from 1000 replicates.

## Supporting Information

Figure S1 **Sequence alignment of DUF3233 representative sequences.** DUF3233 has an average 23 amino acid N-terminal signal sequence (gray) which guides inner membrane translocation. The signal peptidase I cleavage site is marked by a red arrow. Cps_*Colwellia psychrerythraea* (YP_269983.1), Vfi_*Vibrio fischeri* (YP_206466.1), Vpa_*Vibrio parahaemolyticus* (NP_800436.1), Vvu_*Vibrio vulnificus* (NP_762291.1), Vch_*Vibrio cholerae* (NP_232949.1), Vsp_*Vibrio splendidus* (YP_002395311.1), Sba_*Shewanella baltica* (YP_001052604.1), Slo_*Shewanella loihica* (YP_001095898.1), Spi_*Shewanella piezotolerans* (YP_002312093.1), Fba_*Ferrimonas balearica* (YP_003912385.1).
(TIF)

Table S1 **List of DUF3233 homologous sequences.** Sequence search by HHsenser (permissive) picked gram-negative and cyanobacterial (Cyn) hypothetical proteins that share significant homology with DUF3233. These hypothetical proteins posses sequence characteristics that apparently resemble the autotransporter beta-domain. **#** Insignificant Pfam A hits, **$** Significant Pfam A hits.
(DOC)

Table S2 **List of representative sequences used for phylogeny analysis.** C-terminal translocation unit sequences of Autotransporter (Va) [18], Two partner secretion (Vb) [19] proteins and DUF3233 representatives used for phylogenetic analysis.
(DOC)
