# Supplementary figures and images for: Protein Domain of Unknown Function 3233 is a Translocation Domain of Autotransporter Secretory Mechanism in Gamma proteobacteria
Source: PLoS One. 2011 Nov 1;6(11):e25570. doi: 10.1371/journal.pone.0025570 (PMC3206015; doi:10.1371/journal.pone.0025570)

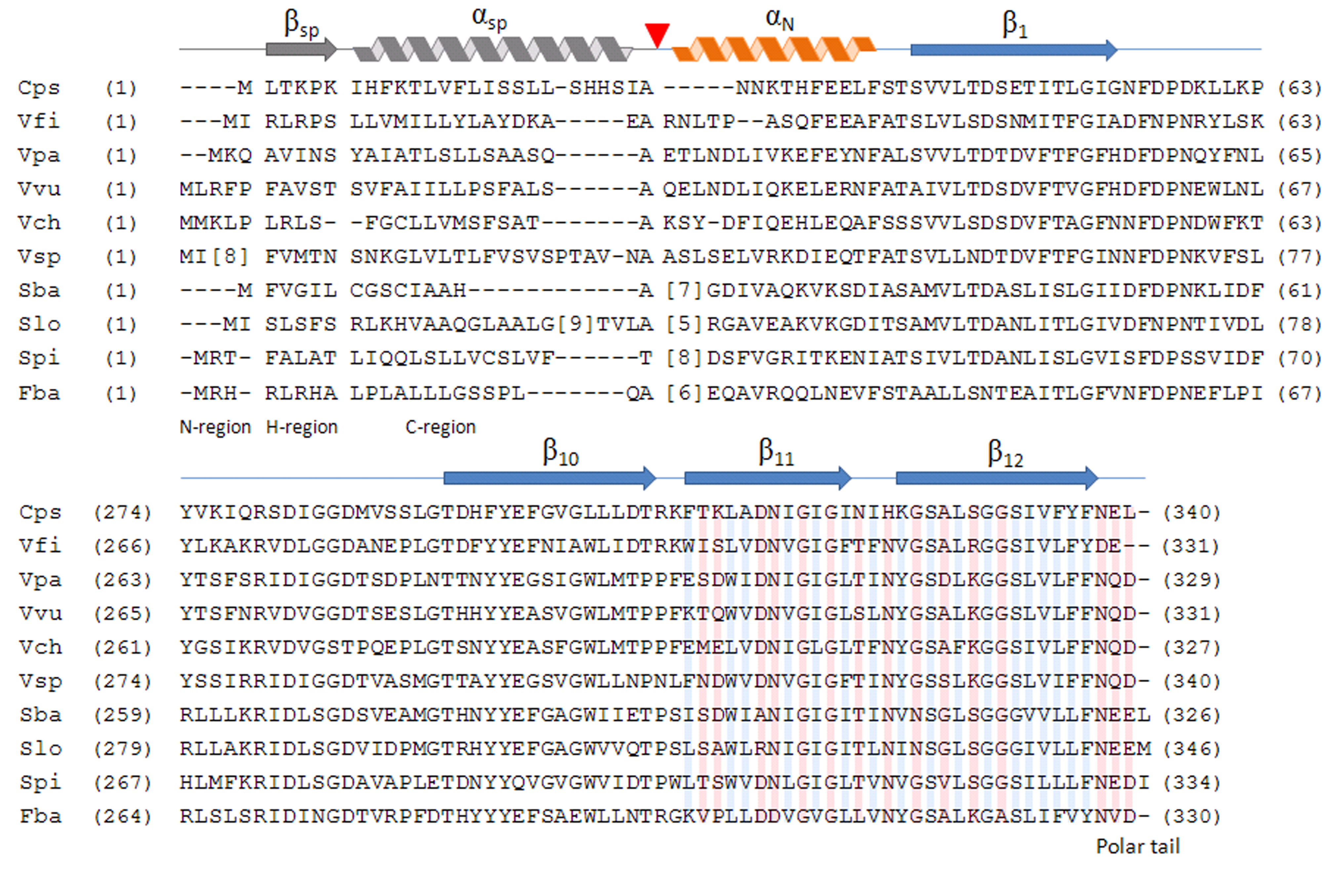

Supplement: Figure S1 — Sequence alignment of DUF3233 representative sequences. DUF3233 has an average 23 amino acid N-terminal signal sequence (gray) which guides inner membrane translocation. The signal peptidase I cleavage site is marked by a red arrow. Cps_Colwellia psychrerythraea (YP_269983.1), Vfi_Vibrio fischeri (YP_206466.1), Vpa_Vibrio parahaemolyticus (NP_800436.1), Vvu_Vibrio vulnificus (NP_762291.1), Vch_Vibrio cholerae (NP_232949.1), Vsp_Vibrio splendidus (YP_002395311.1), Sba_Shewanella baltica (YP_001052604.1), Slo_Shewanella loihica (YP_001095898.1), Spi_Shewanella piezotolerans (YP_002312093.1), Fba_Ferrimonas balearica (YP_003912385.1). (TIF) [file pone.0025570.s001.tif]
