# Supplementary material for: Protein Domain of Unknown Function 3233 is a Translocation Domain of Autotransporter Secretory Mechanism in Gamma proteobacteria
Source: PLoS One. 2011 Nov 1;6(11):e25570. doi: 10.1371/journal.pone.0025570 (PMC3206015; doi:10.1371/journal.pone.0025570)
Supplement: Table S1 — List of DUF3233 homologous sequences. Sequence search by HHsenser (permissive) picked gram-negative and cyanobacterial (Cyn) hypothetical proteins that share significant homology with DUF3233. These hypothetical proteins posses sequence characteristics that apparently resemble the autotransporter beta-domain. # Insignificant Pfam A hits, $ Significant Pfam A hits. (DOC) [file pone.0025570.s002.doc]

| **gid** | **Protein Description** | **Organism** | **Type** | **HHsenser**  **E-value, id%** | **Pfam Family**^#^ |
| --- | --- | --- | --- | --- | --- |
| 268593443 | Hypothetical protein PROVRETT_09930 | P. rettgeri | γ | 1E-136, 84% | Toluene_X |
| 73542925 | Hypothetical protein Reut_A3241 | R. eutropha | β | 1E-103, 35% | Autotransporter |
| 293605291 | Conserved hypothetical protein | A. piechaudii | β | 1E-101, 36% | Autotransporter |
| 145298644 | Hypothetical protein ASA_1655 | A. salmonicida | γ | 3E-98, 32% | Autotransporter**^$^** |
| 311279569 | Hypothetical protein Entcl_2263 | E. cloacae | γ | 1E-97, 34% | Autotransporter |
| 288935374 | Hypothetical protein Kvar_2511 | K. variicola | γ | 3E-97, 34% | Autotransporter |
| 170690261 | Conserved hypothetical protein | B. graminis | β | 9E-91, 19% | Autotransporter |
| 186471209 | Hypothetical protein Bphy_6451 | B. phymatum | β | 5E-88, 20% | Autotransporter |
| 94310634 | Hypothetical protein Rmet_1694 | C. metallidurans | β | 5E-88, 20% | Autotransporter |
| 311107004 | Hypothetical protein AXYL_03822 | A. xylosoxidans | β | 3E-83, 20% | Autotransporter |
| 194288806 | Conserved hypothetical protein | C. taiwanensis | β | 6E-53, 14% | Autotransporter |
| 33601714 | Hypothetical protein BB2738 | B. bronchiseptica | β | 8E-41, 36% | Autotransporter |
| 117621001 | Hypothetical protein AHA_2719 | A. hydrophila | γ | 9E-39, 13% | Autotransporter |
| 15964814 | Hypothetical protein SMc02406 | S. meliloti | α | 7E-27, 20% | Autotransporter |
| 282899355 | Hypothetical protein CRC_00805 | C. raciborskii | Cyn | 1E-24, 15% | GDSL-like Lipase/Acylhydrolase**^$^**  Autotransporter **^$^** |

**Table S1:** **List of DUF3233 homologous sequences**. Sequence search by HHsenser (permissive) picked gram-negative and cyanobacterial (Cyn) hypothetical proteins that share significant homology with DUF3233. These hypothetical proteins posses sequence characteristics that apparently resemble the autotransporter beta-domain.

**#** Insignificant Pfam A hits, **$** Significant Pfam A hits.
