# Supplementary material for: Protein Domain of Unknown Function 3233 is a Translocation Domain of Autotransporter Secretory Mechanism in Gamma proteobacteria
Source: PLoS One. 2011 Nov 1;6(11):e25570. doi: 10.1371/journal.pone.0025570 (PMC3206015; doi:10.1371/journal.pone.0025570)
Supplement: Table S2 — List of representative sequences used for phylogeny analysis. C-terminal translocation unit sequences of Autotransporter (Va) [18], Two partner secretion (Vb) [19] proteins and DUF3233 representatives used for phylogenetic analysis. (DOC) [file pone.0025570.s003.doc]

| Autotransporters |  |  | |  |
| --- | --- | --- | --- | --- |
|  | Description | gid | Organism | |
| Cluster 1 |  |  |  | |
| SSp Sma | Extracellular serine protease | 131087 | Serratia marcescens | |
| Ssp-h1 Sma | Subtilase serine protease | 3688585 | Serratia marcescens | |
| Ssp-h2 Sma | Subtilase serine protease | 1100764 | Serratia marcescens | |
| PspA Pfl | Serine protease homologue | 4115628 | Pseudomonas fluorescens | |
| PspB Pfl | Serine protease homologue | 4115629 | Pseudomonas fluorescens | |
| Ssa1 Mha | Serotype-1-specific antigen | 507431 | Mannheimia haemolytica | |
| SphB1 Bpe | Autotransporter subtilisin-like protease | 14715185 | Bordetella pertussis | |
| NaIP Nme | Neisserial autotransported lipoprotein | 25140440 | Neisseria meningitidis | |
|  |  |  |  | |
| Cluster 2 |  |  |  | |
| VacA Hpy | Vacuolating cytotoxin autotransporter | 2499107 | Helicobacter pylori | |
|  |  |  |  | |
| Cluster 3 and 4 |  |  |  | |
| Aida-1 Eco | AIDA-I autotransporter | 543788 | Escherichia coli | |
| IcsA Sfl | Invasion protein | 13449130 | Shigella flexneri | |
| MisL Sen | MisL protein | 4324610 | Salmonella enterica | |
| TibA Eco | TibA protein | 5305639 | Escherichia coli | |
| Ag43 Eco | Antigen 43 | 54040656 | Escherichia coli | |
| ShdA Sen | ShdA protein | 5107805 | Salmonella enterica | |
| AutA Nme | Autotransporter A | 7649687 | Neisseria meningitidis | |
|  |  |  |  | |
| Cluster 5 |  |  |  | |
| Tsh Eco | Tsh protein | 469236 | Escherichia coli | |
| SepA Sfl | Extracellular serine protease | 12329055 | Shigella flexneri | |
| EspC Eco | EspC protein | 1764164 | Escherichia coli | |
| EspP Eco | Putative exoprotein-precursor | 2244638 | Escherichia coli | |
| Pet Eco | Pet precursor | 3095184 | Escherichia coli | |
| Pic Eco | Pic serine protease precursor | 4574220 | Escherichia coli | |
| SigA Sfl | Exported serine protease | 7682555 | Shigella flexneri | |
| Sat Eco | Secreted autotransporter toxin | 11096073 | Escherichia coli | |
| Vat Eco | Vacuolating autotransporter toxin | 34099063 | Escherichia coli | |
| EpeA Eco | Autotransporter protease | 16417609 | Escherichia coli | |
| EatA Eco | Secreted autotransporter protein | 27549252 | Escherichia coli | |
| EaaA Eco | Serine protease autotransporter | 218690192 | Escherichia coli | |
| EaaC Eco | EaaC protein | 7523532 | Enterobacteria phage | |
| Hbp Eco | Hemoglobin-binding protease | 294492172 | Escherichia coli | |
|  |  |  |  | |
| Cluster 6 |  |  |  | |
| Pertactin Bpe | Pertactin autotransporter | 464364 | Bordetella pertussis | |
| BrkA Bpe | BrkA protein | 562026 | Bordetella pertussis | |
| Tcf Bpe | Tracheal colonization factor protein | 34809494 | Bordetella pertussis | |
| Vag8 Bpe | Vag8 protein | 2997419 | Bordetella pertussis | |
|  |  |  |  | |
| Cluster 7 |  |  |  | |
| PmpD Ctr | Probable outer membrane protein | 14195037 | Chlamydia trachomatis | |
| Pmp20 Cpn | Probable outer membrane protein | 14195024 | Chlamydophila pneumoniae | |
| Pmp21 Cpn | Probable outer membrane protein | 14195023 | Chlamydophila pneumoniae | |
|  |  |  |  | |
| Cluster 8 |  |  |  | |
| App Nme | Adhesion penetration protein | 11071863 | Neisseria meningitidis | |
| IgA1 Hin | Immunoglobulin A1 protease | 1170517 | Haemophilus influenzae | |
| Hap2 Hin | Adhesion and penetration protein | 1170167 | Haemophilus influenzae | |
|  |  |  |  | |
| Cluster 9 |  |  |  | |
| rOmpA Rri | Outer membrane protein A | 112710 | Rickettsia rickettsii | |
| rOmpB Rri | Outer membrane protein B | 6685726 | Rickettsia rickettsii | |
|  |  |  |  | |
| Cluster 10 |  |  |  | |
| ApeE Sen | Outer membrane esterase | 2896133 | Salmonella enterica | |
| EstA Pae | Lipase/esterase | 2218156 | Pseudomonas aeruginosa | |
| Lip-1 Plu | Lipase 1 | 729942 | Photorhabdus luminescens | |
| McaP Mca | McaP protein | 33150472 | Moraxella catarrhalis | |
|  |  |  |  | |
| Cluster 11 |  |  |  | |
| BabA2 Hpy | Adhesin binding fucosylated histo-blood group antigen | 2804778 | Helicobacter pylori | |
| SabA Hpy | Putative Outer membrane protein | 4155217 | Helicobacter pylori | |
| AlpA Hpy | Adhesin | 2344807 | Helicobacter pylori | |
|  |  |  |  | |
| Unassigned |  |  |  | |
| Aae Aac | Adhesin | 30230642 | Aggregatibacter actinomycetemcomitans | |
| NanB Pmu | Sialidase | 11464736 | Pasteurella multocida | |
|  |  |  |  | |
| **DUF3233** |  |  |  | |
| DUF3233 Vch | Hypothetical protein VCA0559 | 15601318 | *Vibrio cholerae* | |
| DUF3233 Cps | Hypothetical protein CPS_3307 | 71277893 | *Colwellia psychrerythraea* | |
| DUF3233 Slo | Hypothetical protein Shew_3773 | 127514701 | *Shewanella loihica* | |
|  |  |  |  | |
| Two Partner Secretion System | | | | |
|  |  |  |  | |
| Cluster1 |  |  |  | |
| FhaC Xor | Outer membrane hemolysin activator protein | 188578848 | Xanthomonas oryzae | |
| Omp Xfa | Outer membrane hemolysin activator protein | 15839139 | Xylella fastidiosa | |
| Omp87 Pmu | Omp87 protein | 27527748 | Pasteurella multocida | |
| Omp Vch | Hypothetical protein VC2548 | 15642543 | Vibrio cholerae | |
|  |  |  |  | |
| Cluster2 |  |  |  | |
| Omp Cmu | Outer membrane protein, putative | 301336699 | Chlamydia muridarum | |
| Omp85 Ctr | OMP85 family membrane protein | 15604962 | Chlamydia trachomatis | |
| Omp85 Cpn | OMP85 family membrane protein | 15618220 | Chlamydophila pneumoniae | |
|  |  |  |  | |
| Cluster3 |  |  |  | |
| Omp1 Bme | Outer membrane protein1 | 265995070 | Brucella melitensis | |
| Omp85 Ngo | Outer membrane protein Omp85 | 291042819 | Neisseria gonorrhoeae | |
| Omp85 Nme | Outer membrane protein OMP85 | 218767125 | Neisseria meningitidis | |
| Oma Plu | Outer membrane antigen | 5689866 | Photorhabdus luminescens | |
| Omp Mlo | Outer membrane protein | 13470835 | Mesorhizobium loti | |
| Omp Aae | Outer membrane protein | 15606510 | Aquifex aeolicus | |
| Omp Dra | Outer membrane protein | 15805407 | Deinococcus radiodurans | |
| Omp85 Cje | Outer membrane protein | 315123731 | Campylobacter jejuni | |
| Omp Pae | Hypothetical protein PA2543 | 15597739 | Pseudomonas aeruginosa | |
| Omp85 Ssp | OMP85 family outer membrane protein | 170076946 | Synechococcus sp. | |
|  |  |  |  | |
| Cluster4 |  |  |  | |
| HpmB Pmi | Hemolysin activator protein | 197285905 | Proteus mirabilis | |
| ShlB Sma | Hemolysin transporter protein | 123205 | Serratia marcescens | |
| HhdB Hdu | HhdB precursor | 1151071 | Haemophilus ducreyi | |
| LspB Hdu | LspB protein | 3929022 | Haemophilus ducreyi | |
| FhaC Bpe | FhaC protein | 163258633 | Bordetella petrii | |
| HecB Nme | Hemolysin activation protein HecB, putative | 161869438 | Neisseria meningitidis | |
| EthB Eta | Activation/secretion protein | 2244626 | Edwardsiella tarda | |
| HlyB Spr | Hemolysin activator HlyB domain-containing protein | 157372714 | Serratia proteamaculans | |

**Table S2: List of representative sequences used for phylogeny analysis.** C-terminal translocation unit sequences of Autotransporter (Va) [18], Two partner secretion (Vb) [19] proteins and DUF3233 representatives used for phylogenetic analysis.
